# Association of Blood Pressure Indices with Right and Left Cardio-Ankle Vascular Index (CAVI) and Its Mathematically Corrected Form (CAVI_0_) for the Evaluation of Atherosclerosis

**DOI:** 10.3390/jpm12091386

**Published:** 2022-08-26

**Authors:** Tzu-Jen Hung, Nan-Chen Hsieh, Elaheh Alizargar, Chyi-Huey Bai, Kai-Wei K. Wang, Shahrokh Hatefi, Javad Alizargar

**Affiliations:** 1 Department of Health Care Management, National Taipei University of Nursing and Health Sciences, Taipei 112, Taiwan; 2 Positron Tomography Center, Shin Kong Wu Ho-Su Memorial Hospital, Taipei 111, Taiwan; 3 Department of Information Management, National Taipei University of Nursing and Health Sciences, Taipei 112, Taiwan; 4 Institute of Public Health, National Yang Ming Chiao Tung University, Taipei 112, Taiwan; 5 School of Public Health, College of Public Health, Taipei Medical University, Taipei 11031, Taiwan; 6 School of Nursing, National Taipei University of Nursing and Health Sciences, Taipei 112, Taiwan; 7 Ultra-High Precision Manufacturing Laboratory, Department of Mechatronics Engineering, Faculty of Engineering, The Built Environment and Technology, Nelson Mandela University, Port Elizabeth 6000, South Africa; 8 Research Center for Healthcare Industry Innovation, National Taipei University of Nursing and Health Sciences, Taipei 112, Taiwan

**Keywords:** atherosclerosis, cardiovascular diseases, cardio-ankle vascular index, ankle brachial index, hypertension, risk factor

## Abstract

Background and Objectives: The cardio-ankle vascular index (CAVI) is an index for arterial stiffness that is theoretically independent of blood pressure. CAVI_0_ is the mathematically corrected formula of CAVI that has been claimed to be less dependent on blood pressure changes. The association of right and left CAVI and CAVI_0_ with blood pressure indices can reveal the importance of considering the blood pressure of the patient despite their theoretical independency. In this study, we assessed the right and left CAVI and CAVI_0_ and evaluated the main effect of blood pressure indices on them with respect to age. Materials and Methods: We collected the following data of 136 community-dwelling individuals: age; sex; weight; height; body mass index; right and left CAVI and CAVI_0_; and systolic (SBP), diastolic (DBP), and mean blood pressure (MBP). The association of right and left CAVI and CAVI_0_ with blood pressure indices was evaluated using regression analysis. Results: Regression analysis revealed that SBP, DBP, and MBP were associated with right and left CAVI (independent of age). Moreover, SBP was associated with both right and left CAVI_0_ (independent of age), whereas DBP was not associated with right or left CAVI_0_. Conclusion: Right and left arterial stiffness measured using CAVI has no different associations with SBP, DBP, and MBP. Both right and left CAVI_0_ were independently associated with SBP, whereas neither left nor right CAVI_0_ was independently associated with DBP. MBP was only associated with the right-side CAVI_0_ in community-dwelling individuals.

## 1. Introduction

Atherosclerosis is a thrombotic disease that can remain asymptomatic until the late stages of life. Thrombosis includes embolization and vascular occlusions, which can have life-threatening manifestations such as myocardial infarction (MI) and stroke [1]. Arteriosclerosis and atherosclerosis have two different definitions, but atherosclerosis is a specific type of arteriosclerosis [2]. Arterial stiffness indicates the extent of arteriosclerosis and is believed to be a reliable predictor of events associated with cardiovascular diseases (CVDs) [3]. Arterial stiffness indices are cost-effective screening tests that are useful for determining arterial health and future CVD events, especially in mass screening programs and large-population studies [2]. Pulse wave velocity (PWV) has become widely used globally as a surrogate marker of arterial stiffness [3]. Other indices of arterial stiffness are carotid–femoral PWV and the ankle–brachial index. However, all these indices are affected by changes in blood pressure (BP) during measurement [4,5]. To resolve this issue, the cardio-ankle vascular index (CAVI), which is believed to be independent of BP during measurement, was proposed by Shirai et al. in 2006 and has since been used by researchers [6]. CAVI can be calculated by a reference device named VaSera VS-1500 and a new version of it (VsSera-2000). Spronck et al. mathematically corrected the CAVI formula (named CAVI_0_) and claimed that this new index is more independent of blood pressure [7]. CAVI and CAVI_0_ are calculated based on Equations (1) and (2):

CAVI = a[(2ρ/ΔP) × ln(SBP/DBP)PWV^2^] + b (1)

CAVI_0_ = 2ρ × (PWV^2^/DBP) − ln (DBP/P_0_) (2)

where SBP and DBP are systolic and diastolic BP, ΔP = SBP − DBP, ρ is the blood density, a and b are the constants automatically measured using the device to match the aortic PWV, and P_0_ is the reference pressure (100 mmHg).

The claims of BP independency of CAVI and CAVI_0_ can be misleading. BP as a risk factor for arterial stiffness always plays a vital role in CAVI and CAVI_0_ measurements [8,9]. Factors associated with CAVI and CAVI_0_ can lead clinicians to set up a protocol for screening individuals at risk of life-threatening events, such as MI and stroke, by detecting patients with high arterial stiffness.

Right and left arterial stiffness measurements can be different, as the arterial tree is different on the right and left sides. At present, the average of left and right arterial stiffness is used to measure the overall arterial stiffness in an individual. However, no study has evaluated the factors associated with the right and left CAVI and CAVI_0_. Our previous study reported that age is the main independent determinant of overall CAVI and CAVI_0_ and that BP indices are not an important independent determinant of CAVI and CAVI_0_ [10]. In this study, we focused on right and left CAVI and CAVI_0_ separately and examined the main effect of BP on them with respect to age.

## 2. Materials and Methods

We retrieved and analyzed the data of 163 community-dwelling individuals recruited for annual checkups for a community-based prospective cohort study in Taipei. Details of the study protocol and data collection have been published elsewhere [9]. In summary, participants who were ≥30 years old, had complete questionnaires, and had no history of cancer or chronic kidney disease underwent CAVI measurement with VaSera VS-1000 (Fukuda Denshi, Tokyo, Japan). CAVI_0_ was calculated using the tool provided by Spronck et al. [11]. This study was conducted in accordance with the Declaration of Helsinki, and the protocol was approved by the Ethics Committee of Taipei Medical University and the Institutional Review Board (reference numbers 94E-183, 94E-198, and 96E-004). This study is cross-sectional despite the fact that the data were obtained from a prospective cohort study. Before participation in this study, all patients provided informed consent for inclusion in this study.

The patients’ age, sex, weight, height, body mass index (BMI; calculated as weight (kg)/height^2^ (m^2^)), systolic BP (SBP), diastolic BP (DBP), mean BP (MBP), and right and left CAVI and CAVI_0_ were extracted from the database. Continuous data was presented as mean ± standard deviation. The Shapiro–Wilk test was used for the test for normality. Univariate and multivariate linear and logistic regression was performed to assess the association of blood pressure indices and CAVI/CAVI_0_. Different sets of regression were used for the right and left CAVI and CAVI_0_. As the cut-off value for CAVI and CAVI_0_ has not been evaluated in the literature, 75 percentiles of CAVI and CAVI_0_ were used to determine high and low CAVI and CAVI_0_ for the logistic regression. The odds ratio and confidence interval (CI) were then calculated. Assumptions of the linear and logistic regression were checked for each model separately using the Box-Tidwell test, variance inflation factor (VIF), tolerance and residuals by regressors for the independence of errors, Durbin–Watson test for autocorrelation and homoscedasticity, and the scatter plot to check whether the data were homoscedastic (residuals are equal across the regression line). All statistical analyses were performed using SAS 9.4 (SAS, Cary, NC, USA).

## 3. Results

The descriptive characteristics of the study individuals have been reported previously [10]. In brief, the participants included 98 (60.12%) women and 65 (39.88%) men, with a mean age of 63.07 ± 9.40 years. The descriptive distribution analysis for CAVI and CAVI_0_ is presented in Table 1.

We used 75 percentiles of mean CAVI to set the high and low values of right, left, and mean CAVI and CAVI_0_. The distribution of the age and BP indices based on this classification for CAVI and CAVI_0_ is presented in Table 2; Table 3, respectively.

The results of the assumptions checking for the linear and logistic regression are shown in the Supplementary Materials (Tables S1–S38 and Figures S1–S33). 

The results of the linear regression for the CAVI and CAVI_0_ using the age and blood pressure indices separately for the left and right side are shown in Table 4 and Table 5.

Univariate logistic regressions using high CAVI and CAVI_0_ (of the right and left sides separately, using 75 percentiles of these indices) as the dependent variables and BP indices as independent variables were performed. We increased the model complexity by adding age as an independent variable to every model to assess the independency of the BP indices with respect to age, thus forming multivariate logistic regression models. The results of these regression analyses are presented in Table 6 and Table 7.

## 4. Discussion

The results revealed that SBP, DBP, and MBP were associated with right and left CAVI (independent of age). Moreover, SBP was associated with both right and left CAVI_0_ (independent of age), but DBP was not associated with either right or left CAVI_0_. MBP had a significant independent association with right but not left CAVI_0_.

CAVI is an index of arterial stiffness from the aorta to the ankles. Higher BP during measurement results in higher PWV because of arterial elasticity. CAVI is intended to correct this dependency by canceling the effects of BP on PWV [10]. Its formula was modified by Spronck et al. to cancel the BP effects even further [7]. However, our results suggest that patients’ BP is still associated with CAVI and CAVI_0_. This does not mean these indices are not effective in canceling the effects of BP on arterial stiffness because higher BP is also a risk factor for stiffer arteries. Therefore, this study further supported the notion that BP is an associated factor when dealing with arterial stiffness.

The results of the linear regression in our study revealed that the right and left CAVI was associated with BP indices. However, in contrast, logistic regression showed that high right CAVI but not left CAVI is associated with BP indices. Although logistic regression might lose its power by dichotomization of the dependent variable (CAVI), association of blood pressure indices with high CAVI might be a point of interest to some researchers. In our study, BP was measured on the patients’ left side as the standard method for measuring BP if only one side is going to be used as a reference. The Framingham Heart Study on 3390 participants indicated a 3-mmHg difference in the median BP between the right and left arms [12]; our result of the observed difference in the association of right and left CAVI with BP indices is unlikely to be caused by this difference. The left CAVI is affected by the anatomy of the heart and large vessel differences between the right and left. Therefore, we calculated the arterial stiffness indices of the left and right sides separately. Although after considering the results of the logistic regression, SBP was independently associated with both right and left CAVI_0_, whereas CAVI was associated only with right CAVI; thus, CAVI may not necessarily be superior to CAVI_0_ in eliminating the effect of BP changes on arterial stiffness measurements. However, our data indicate that when measuring CAVI_0_, SBP should be considered as an independent determinant factor that influences both right and left CAVI_0_. However, the right-side CAVI may be a more reliable index for arterial stiffness given its lesser dependency on BP changes.

Our study has some limitations. First, the cross-sectional design precluded the determination of causality. Second, we did not find a strong difference between the association of the right and left CAVI and CAVI_0_ with BP indices, except for MBP, which was only associated with right not left CAVI_0_. We should mention that only age was used to control as a confounder, but other confounders can still exist, and residual confounding is still an issue that might explain the absence of a statistical difference. However, our results did suggest a difference in the nature of the influential characteristics of BP on right and left CAVI and CAVI_0_. Finally, we only had left BP data for the study participants, which made the interpretations of the study results more challenging. Moreover, a very important study limitation is the arbitrary choice of the cut-off point for CAVI and CAVI_0_. Future case–control studies or randomized control trials might help to determine the causal effects of BP on arterial stiffness. 

Nevertheless, our study had certain strengths, such as separating right and left CAVI and CAVI_0_ and considering the age of the participants as the main influential factor in arterial stiffness, considering the results of our previous study. 

## 5. Conclusions

Right and left CAVI did not have a different association with SBP, DBP, and MBP. Although, high CAVI on the right and left sides (based on the logistic regression results) had different associations with SBP, DBP, and MBP: only the high right CAVI was independently associated with these three indices, making the left CAVI more reliable for evaluation of high arterial stiffness, especially for the identification of patients with high arterial stiffness.

Both right and left CAVI_0_ were independently associated with SBP, whereas neither left nor right CAVI_0_ were independently associated with DBP. MBP was only associated with right-side CAVI_0_ in community-dwelling individuals. These associations should be verified in future studies and considered when interpreting the results of arterial stiffness measurements in community-dwelling individuals to identify individuals at high risk of serious disease due to arterial stiffness.

## Figures and Tables

**Table 1:** Descriptive distribution analysis for CAVI and CAVI_0_.

	CAVI			CAVI_0_		
	Mean	Left	Right	Mean	Left	Right
75 Percentile (Q3)	9.45	9.3	9.5	15.22	14.90	15.36
50 Percentile (median)	8.75	8.8	8.7	13.17	13.18	13.24
25 Percentile (Q1)	7.95	7.9	8.0	11.40	11.20	11.36

CAVI = cardio-ankle vascular index; CAVI_0_ = mathematically corrected cardio-ankle vascular index.

**Table 2:** Distribution of age and blood pressure indices based on high and low values of right, left, and mean CAVI.

	Mean ± SD	CAVI								
		Right			Left			Mean		
		High	Low	*p* * Value	High	Low	*p* Value	High	Low	*p* Value
Age	63.07 ± 9.40	69.86 ± 6.72	60.47 ± 8.99	**<0.001**	69.57 ± 7.06	60.27 ± 8.90	**<0.001**	69.73 ± 7.04	60.82 ± 9.04	**<0.001**
SBP	130.10 ± 17.37	137.24 ± 18.44	127.38 ± 16.22	**0.001**	133.36 ± 19	128.70 ± 16.51	0.116	136.60 ± 19.05	127.91 ± 16.28	**0.005**
DBP	80.74 ± 10.40	82.93 ± 10.60	79.90 ± 10.24	0.097	80.93 ± 9.67	80.65 ± 10.74	0.875	82.24 ± 10.34	80.23 ± 10.41	0.286
MBP	97.19 ± 12.04	101.03 ± 12.51	95.73 ± 11.57	**0.011**	98.41 ± 12.20	96.67 ± 11.98	0.398	100.36 ± 12.65	96.13 ± 11.69	0.051

CAVI = cardio-ankle vascular index; SBP = systolic blood pressure; DBP = diastolic blood pressure; MBP = mean blood pressure. * ANOVA test. SD = standard deviation; *p* values < 0.05 are in bold.

**Table 3:** Distribution of age and blood pressure indices based on high and low values of right, left, and mean CAVI_0_.

Mean ± SD	CAVI_0_								
	Right			Left			Mean		
	High	Low	*p* * Value	High	Low	*p* Value	High	Low	*p* Value
Age	69.75 ± 6.73	60.81 ± 9.11	**<0.001**	70.70 ± 6.64	60.50 ± 8.79	<0.001	70.26 ± 6.86	60.64 ± 8.90	**<0.001**
SBP	139.21 ± 18.66	127.04 ± 15.86	**<0.001**	138.39 ± 18.03	127.31 ± 16.30	<0.001	139.29 ± 18.53	127.01 ± 15.89	**<0.001**
DBP	81.65 ± 10.80	80.43 ± 10.29	0.516	80.82 ± 9.72	80.71 ± 10.66	0.950	82.09 ± 10.67	80.28 ± 10.31	0.336
MBP	100.84 ± 12.86	95.96 ± 11.55	**0.024**	100.01 ± 11.83	96.24 ± 12.01	0.083	101.16 ± 12.66	95.86 ± 11.57	**0.014**

CAVI_0_ = mathematically corrected cardio-ankle vascular index; SBP = systolic blood pressure; DBP = diastolic blood pressure; MBP = mean blood pressure. * ANOVA test. SD = standard deviation; *p* values < 0.05 are in bold.

**Table 4:** Linear regression models to assess the independency of the association between blood pressure indices and CAVI.

Models		CAVI					
		Right			Left		
		Regression Beta Coefficient	Standard Error	*p* Value	Regression Beta Coefficient	Standard Error	*p* Value
SBP		0.020	0.004	**<0.001**	0.017	0.004	**<0.001**
SBP + age	SBP	0.012	0.004	**0.002**	0.010	0.004	**0.015**
	Age	0.063	0.007	**<0.001**	0.060	0.007	**<0.001**
DBP		0.022	0.008	**0.008**	0.017	0.008	**0.036**
DBP + age	DBP	0.021	0.006	**0.001**	0.017	0.006	**0.012**
	Age	0.068	0.007	**<0.001**	0.064	0.007	**<0.001**
MBP		0.025	0.007	**<0.001**	0.020	0.006	**0.003**
MBP + age	MBP	0.019	0.005	**0.001**	0.015	0.005	**0.009**
	Age	0.065	0.007	**<0.001**	0.062	0.007	**<0.001**

CAVI = cardio-ankle vascular index; SBP = systolic blood pressure; DBP = diastolic blood pressure; MBP = mean blood pressure; CI = confidence interval; *p* values < 0.05 are in bold.

**Table 5:** Linear regression models to assess the independency of the association between blood pressure indices and CAVI_0_.

Models		CAVI_0_					
		Right			Left		
		Regression Beta Coefficient	Standard Error	*p* Value	Regression Beta Coefficient	Standard Error	*p* Value
SBP		0.054	0.011	**<0.001**	0.047	0.011	**<0.001**
SBP + age	SBP	0.034	0.009	**<0.001**	0.028	0.009	**0.004**
	Age	0.166	0.018	**<0.001**	0.158	0.017	**<0.001**
DBP		0.021	0.020	0.312	0.008	0.020	0.663
DBP + age	DBP	0.020	0.016	0.215	0.008	0.016	0.612
	Age	0.180	0.018	**<0.001**	0.169	0.017	**<0.001**
MBP		0.048	0.017	**0.007**	0.037	0.017	**0.032**
MBP + age	MBP	0.033	0.014	**0.019**	0.022	0.013	0.102
	Age	0.175	0.018	**<0.001**	0.166	0.017	**<0.001**

CAVI_0_ = mathematically corrected cardio-ankle vascular index; SBP = systolic blood pressure; DBP = diastolic blood pressure; MBP = mean blood pressure; *p* values < 0.05 are in bold.

**Table 6:** Logistic regression models to assess the independency of the association between blood pressure indices and CAVI.

Models		CAVI							
		Right				Left			
		Beta Coefficient	*p* Value	OR	CI	Beta Coefficient	*p* Value	OR	CI
SBP		0.033	**0.001**	1.034	1.013–1.056	0.015	0.117	1.016	0.996–1.036
SBP + age	SBP	0.027	**0.026**	1.027	1.003–1.052	0.003	0.752	1.004	0.982–1.06
	Age	0.132	**<0.001**	1.142	1.081–1.206	0.135	**<0.001**	1.145	1.086–1.206
DBP		0.028	0.098	1.029	0.995–1.064	0.002	0.874	1.003	0.971–1.035
DBP + age	DBP	0.041	**0.049**	1.042	1.000–1.086	0.003	0.865	1.003	0.965–1.043
	Age	0.143	**<0.001**	1.155	1.093–1.220	0.136	**<0.001**	1.146	1.088–1.207
MBP		0.037	**0.013**	1.038	1.008–1.070	0.012	0.396	1.012	0.984–1.041
MBP + age	MBP	0.039	**0.027**	1.041	1.004–1.078	0.004	0.801	1.004	0.972–1.038
	Age	0.139	**<0.001**	1.149	1.088–1.214	0.136	**<0.001**	1.146	1.088–1.207

CAVI = cardio-ankle vascular index; SBP = systolic blood pressure; DBP = diastolic blood pressure; MBP = mean blood pressure; CI = confidence interval; *p* values < 0.05 are in bold.

**Table 7:** Logistic regression models to assess the independency of the association between blood pressure indices and CAVI_0_.

Models		CAVI_0_							
		Right				Left			
		Beta Coefficient	*p* Value	OR	CI	Beta Coefficient	*p* Value	OR	CI
SBP		0.042	**<0.001**	1.043	1.020–1.067	0.038	**<0.001**	1.039	1.016–1.062
SBP + age	SBP	0.037	**0.003**	1.039	1.013–1.065	0.032	**0.012**	1.033	1.007–1.060
	Age	0.121	**<0.001**	1.129	1.069–1.192	0.154	**<0.001**	1.167	1.089–1.241
DBP		0.011	0.513	1.011	0.0978–1.046	0.001	0.950	1.001	0.968–1.036
DBP + age	DBP	0.015	0.445	1.016	0.976–1.058	0.001	0.943	1.002	0.960–1.045
	Age	0.128	**<0.001**	**1.137**	**1.079–1.199**	0.159	**<0.001**	1.173	1.105–1.245
MBP		0.034	**0.038**	1.035	1.004–1.067	0.026	0.085	1.027	0.996–1.058
MBP + age	MBP	0.034	0.054	1.035	0.999–1.073	0.024	0.186	1.025	0.988–1.063
	Age	0.127	**<0.001**	1.136	1.077–1.198	0.158	**<0.001**	1.172	1.104–1.245

CAVI_0_ = mathematically corrected cardio-ankle vascular index; SBP = systolic blood pressure; DBP = diastolic blood pressure; MBP = mean blood pressure; CI = confidence interval; *p* values < 0.05 are in bold.

## Data Availability

The data presented in this study are available on request from the corresponding author. The data are not publicly available due to institutional review board statement of Taipei Medical University.

## Supplementary Materials

The following supporting information can be downloaded at: https://www.mdpi.com/article/10.3390/jpm12091386/s1, Figure S1: Fit statistics and assumption checking for the Linear regression, dependent variable CAVI and independent variable SBP; Figure S2: Fit statistics and assumption checking for the Linear regression, dependent variable Right CAVI and independent variable SBP and age; Figure S3: Fit statistics and assumption checking for the Linear regression, dependent variable Right CAVI and independent variable DBP; Figure S4: Fit statistics and assumption checking for the Linear regression, dependent variable Right CAVI and independent variable DBP and age; Figure S5: Fit statistics and assumption checking for the Linear regression, dependent variable Right CAVI and independent variable MBP; Figure S6: Fit statistics and assumption checking for the Linear regression, dependent variable Right CAVI and independent variable MBP and age; Figure S7: Fit statistics and assumption checking for the Linear regression, dependent variable Left CAVI and independent variable SBP; Figure S8: Fit statistics and assumption checking for the Linear regression, dependent variable Left CAVI and independent variable SBP and age; Figure S9: Fit statistics and assumption checking for the Linear regression, dependent variable Left CAVI and independent variable DBP; Figure S10: Fit statistics and assumption checking for the Linear regression, dependent variable Left CAVI and independent variable DBP and age; Figure S11: Fit statistics and assumption checking for the Linear regression, dependent variable Left CAVI and independent variable MBP; Figure S12: Fit statistics and assumption checking for the Linear regression, dependent variable Left CAVI and independent variable MBP and age; Figure S13: Fit statistics and assumption checking for the Linear regression, dependent variable Right CAVI_0_ and independent variable SBP; Figure S14: Fit statistics and assumption checking for the Linear regression, dependent variable Right CAVI_0_ and independent variable SBP and age; Figure S15: Fit statistics and assumption checking for the Linear regression, dependent variable Right CAVI_0_ and independent variable DBP; Figure S16: Fit statistics and assumption checking for the Linear regression, dependent variable Right CAVI_0_ and independent variable DBP and age; Figure S17: Fit statistics and assumption checking for the Linear regression, dependent variable Right CAVI_0_ and independent variable MBP; Figure S18: Fit statistics and assumption checking for the Linear regression, dependent variable Right CAVI_0_ and independent variable MBP and age; Figure S19: Fit statistics and assumption checking for the Linear regression, dependent variable Left CAVI_0_ and independent variable SBP; Figure S20: Fit statistics and assumption checking for the Linear regression, dependent variable Right CAVI_0_ and independent variable SBP and age; Figure S21: Fit statistics and assumption checking for the Linear regression, dependent variable Right CAVI_0_ and independent variable DBP; Figure S22: Fit statistics and assumption checking for the Linear regression, dependent variable Right CAVI_0_ and independent variable DBP and age; Figure S23: Fit statistics and assumption checking for the Linear regression, dependent variable Right CAVI_0_ and independent variable MBP; Figure S24: Fit statistics and assumption checking for the Linear regression, dependent variable Right CAVI_0_ and independent variable MBP and age; Figure S25: Test for the independence of errors assumption, RCAVI = right CAVI, SBP = Systolic Blood Pressure; Figure S26: Test for the independence of errors assumption, RCAVI = right CAVI, DBP = Diastolic Blood Pressure; Figure S27: Test for the independence of errors assumption, RCAVI = right CAVI, MBP = Mean Blood Pressure; Figure S28: Test for the independence of errors assumption, LCAVI = Left CAVI, SBP = Systolic Blood Pressure; Figure S29: Test for the independence of errors assumption, LCAVI = Left CAVI, DBP = Diastolic Blood Pressure; Figure S30: Test for the independence of errors assumption, LCAVI = Left CAVI, MBP = Mean Blood Pressure; Figure S31: Test for the independence of errors assumption, CAVI_0__R = Right CAVI, SBP = Systolic Blood Pressure; Figure S32: Test for the independence of errors assumption, CAVI_0__R = Right CAVI, DBP = Diastolic Blood Pressure; Figure S33: Test for the independence of errors assumption, CAVI_0__R = Right CAVI, MBP = Mean Blood Pressure. Table S1: Test of linearity between continuous variables of the logistic regression analysis, regarding CAVI; Table S2: test of linearity between continuous variables of the logistic regression analysis, regarding CAVI_0_. Table S3: test of autocorrelation for the Linear regression, dependent variable CAVI and independent variable SBP; Table S4: test of autocorrelation for the Linear regression, dependent variable CAVI and independent variable SBP and age; Table S5: Linear regression analysis for the test of collinearity between the independent variables in the regression models, Right CAVI as the dependent variable and SBP and Age as independent ones; Table S6: test of autocorrelation for the Linear regression, dependent variable Right CAVI and independent variable DBP; Table S7: test of autocorrelation for the Linear regression, dependent variable Right CAVI and independent variable DBP and age; Table S8: Linear regression analysis for the test of collinearity between the independent variables in the regression models, Right CAVI as the dependent variable and DBP and Age as independent ones; Table S9: test of autocorrelation for the Linear regression, dependent variable Right CAVI and independent variable MBP; Table S10: test of autocorrelation for the Linear regression, dependent variable Right CAVI and independent variable MBP and age; Table S11: Linear regression analysis for the test of collinearity between the independent variables in the regression models, Right CAVI as the dependent variable and MBP and Age as independent ones, MBP = Mean Blood Pressure; Table S12: test of autocorrelation for the Linear regression, dependent variable Left CAVI and independent variable SBP; Table S13: test of autocorrelation for the Linear regression, dependent variable Left CAVI and independent variable SBP and age; Table S14: Linear regression analysis for the test of collinearity between the independent variables in the regression models, Left CAVI as the dependent variable and SBP and Age as independent ones; Table S15: test of autocorrelation for the Linear regression, dependent variable Left CAVI and independent variable DBP; Table S16: test of autocorrelation for the Linear regression, dependent variable Left CAVI and independent variable DBP and age; Table S17: Linear regression analysis for the test of collinearity between the independent variables in the regression models, Left CAVI as the dependent variable and DBP and Age as independent ones; Table S18: test of autocorrelation for the Linear regression, dependent variable Left CAVI and independent variable MBP; Table S19: test of autocorrelation for the Linear regression, dependent variable Left CAVI and independent variable MBP and age; Table S20: Linear regression analysis for the test of collinearity between the independent variables in the regression models, Left CAVI as the dependent variable and MBP and Age as independent ones; Table S21: Test of autocorrelation for the Linear regression, dependent variable Right CAVI0 and independent variable SBP; Table S22: test of autocorrelation for the Linear regression, dependent variable Right CAVI0 and independent variable SBP and age; Table S23: Linear regression analysis for the test of collinearity between the independent variables in the regression models, Right CAVI0 as the dependent variable and SBP and Age as independent ones, SBP = Systolic Blood Pressure; Table S24: test of autocorrelation for the Linear regression, dependent variable Right CAVI0 and independent variable DBP; Table S25: test of autocorrelation for the Linear regression, dependent variable Right CAVI0 and independent variable DBP and age; Table S26: Linear regression analysis for the test of collinearity between the independent variables in the regression models, Right CAVI0 as the dependent variable and DBP and Age as independent ones, DBP = Diastolic Blood Pressure; Table S27: test of autocorrelation for the Linear regression, dependent variable Right CAVI0 and independent variable MBP; Table S28: test of autocorrelation for the Linear regression, dependent variable Right CAVI0 and independent variable MBP and age; Table S29: Linear regression analysis for the test of collinearity between the independent variables in the regression models, Right CAVI0 as the dependent variable and MBP and Age as independent ones, MBP = Mean Blood Pressure; Table S30: test of autocorrelation for the Linear regression, dependent variable Left CAVI0 and independent variable SBP; Table S31: test of autocorrelation for the Linear regression, dependent variable Left CAVI0 and independent variable SBP and age; Table S32: Linear regression analysis for the test of collinearity between the independent variables in the regression models, Left CAVI_0_ as the dependent variable and SBP and Age as independent ones, SBP = Systolic Blood Pressure; Table S33: test of autocorrelation for the Linear regression, dependent variable Left CAVI_0_ and independent variable DBP; Table S34: test of autocorrelation for the Linear regression, dependent variable Left CAVI_0_ and independent variable DBP and age; Table S35: Linear regression analysis for the test of collinearity between the independent variables in the regression models, Left CAVI_0_ as the dependent variable and DBP and Age as independent ones, DBP = Diastolic Blood Pressure; Table S36: test of autocorrelation for the Linear regression, dependent variable Left CAVI_0_ and independent variable MBP; Table S37: test of autocorrelation for the Linear regression, dependent variable Left CAVI_0_ and independent variable MBP and age; Table S38: Linear regression analysis for the test of collinearity between the independent variables in the regression models, Left CAVI_0_ as the dependent variable and MBP and Age as independent ones, MBP = Mean Blood Pressure.
