# Supplementary material for: Association of Blood Pressure Indices with Right and Left Cardio-Ankle Vascular Index (CAVI) and Its Mathematically Corrected Form (CAVI0) for the Evaluation of Atherosclerosis
Source: J Pers Med. 2022 Aug 26;12(9):1386. doi: 10.3390/jpm12091386 (PMC9501430; doi:10.3390/jpm12091386)
Supplement: Supplementary file 1 [file jpm-12-01386-s001.zip › jpm-1812781-supplementary.pdf]

Table S 1- test of linearity between continuous variables of the logistic regression analysis, regarding CAVI

Models		High CAVI			
		Right		Left	
		Beta coefficient	P value	Beta coefficient	P value
SBP+SBP*Ln(SBP)	SBP	-0.232	0.76	-0.507	0.45
	SBP*Ln(SBP)	0.045	0.72	0.088	0.43
SBP+age+ SBP*Ln(SBP)+Age*Ln(age)	SBP	-0.836	0.37	-1.217	0.14
	Age	2.856	0.20	2.018	0.30
	SBP*Ln(SBP)	0.146	0.36	0.206	0.14
	Age*Ln(age)	-0.523	0.22	-0.361	0.33
DBP+DBP*Ln(DBP)	DBP	-0.038	0.97	1.199	0.31
	DBP*Ln(DBP)	0.012	0.95	-0.221	0.31
DBP+age+Ln(DBP)+Ln(age)	DBP	-2.22	0.14	-0.178	0.89
	Age	3.479	0.13	2.040	0.29
	DBP*Ln(DBP)	0.419	0.13	0.033	0.89
	Age*Ln(age)	-0.63	0.15	-0.367	0.32
MBP+MBP*Ln(SBP)	MBP	-0.424	0.69	0.056	0.95
	MBP*Ln(DBP)	0.082	0.67	-0.007	0.96
MBP+age+Ln(MBP)+Ln(age)	MBP	-2.203	0.12	-1.302	0.29
	Age	3.194	0.16	2.134	0.27
	MBP*Ln(MBP)	0.401	0.11	0.233	0.29
	Age*Ln(age)	-0.585	0.18	-0.384	0.30

Table S 2-test of linearity between continuous variables of the logistic regression analysis, regarding $CAVI_0$

**Models**		**High $CAVI_0$**			
		**Right**		**Left**	
		**Beta coefficient**	**P value**	**Beta coefficient**	**P value**
SBP+SBP*Ln(SBP)	SBP	-0.445	0.592	0.018	0.981
	SBP*Ln(SBP)	0.0825	0.557	0.003	0.981
SBP+age+ SBP*Ln(SBP)+Age*Ln(age)	SBP	-1.107	0.279	-0.631	0.539
	Age	2.953	0.194	2.144	0.392
	SBP*Ln(SBP)	0.193	0.263	0.112	0.518
	Age*Ln(age)	-0.543	0.213	-0.381	0.427
DBP+DBP*Ln(DBP)	DBP	-0.258	0.817	1.046	0.401
	DBP*Ln(DBP)	0.050	0.809	-0.193	0.402
DBP+age+Ln(DBP)+Ln(age)	DBP	-2.326	0.108	-0.626	0.681
	Age	3.707	0.117	2.507	0.319
	DBP*Ln(DBP)	0.433	0.106	0.116	0.680
	Age*Ln(age)	-0.685	0.130	-0.450	0.350
MBP+MBP*Ln(SBP)	MBP	-0.424	0.670	0.454	0.690
	MBP*Ln(DBP)	0.082	0.670	-0.076	0.707
MBP+age+Ln(MBP)+Ln(age)	MBP	-2.437	0.083	-0.908	0.518
	Age	3.393	0.144	2.387	0.340
	MBP*Ln(MBP)	0.441	0.079	0.166	0.507
	Age*Ln(age)	-0.625	0.160	-0.427	0.372

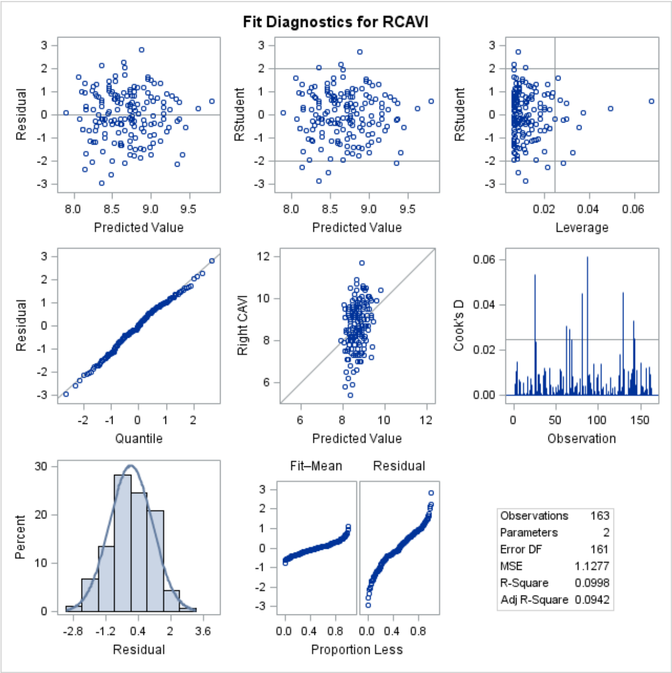

Figure S 1- Fit statistics and assumption checking for the Linear regression, dependent variable CAVI and independent variable SBP

Table S 3- test of autocorrelation for the Linear regression, dependent variable CAVI and independent variable SBP

Durbin-Watson Statistics

Order	DW	Pr < DW	Pr > DW
1	1.8125	0.1145	0.8855

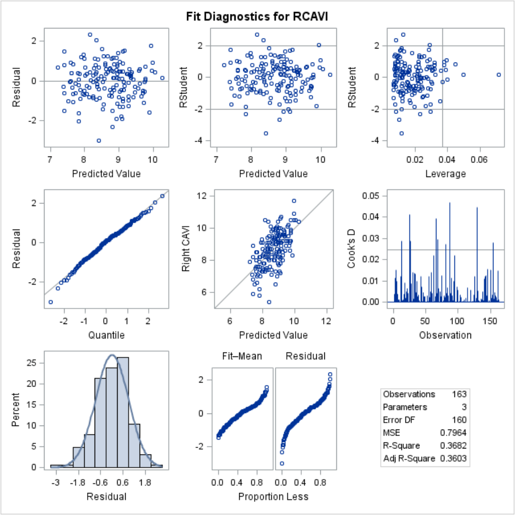

Figure S 2- Fit statistics and assumption checking for the Linear regression, dependent variable Right CAVI and independent variable SBP and age

Table S 4- test of autocorrelation for the Linear regression, dependent variable CAVI and independent variable SBP and age

Durbin-Watson Statistics			
**Order**	**DW**	**Pr < DW**	**Pr > DW**
**1**	2.0082	0.5166	0.4834

Table S 5- Linear regression analysis for the test of collinearity between the independent variables in the regression models, Right CAVI as the dependent variable and SBP and Age as independent ones

Variable	Parameter Estimate	Standard Error	t Value	Pr > |t|	Tolerance	Variance Inflation
**Intercept**	3.04569	0.64226	4.74	<.0001	.	0
**SBP**	0.01276	0.00414	3.09	0.0024	0.95140	1.05109
**Age**	0.06303	0.00764	8.25	<.0001	0.95140	1.05109

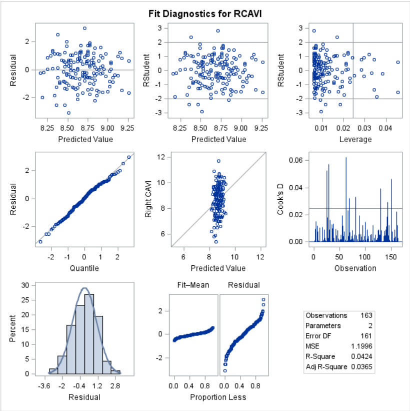

Figure S 3- Fit statistics and assumption checking for the Linear regression, dependent variable Right CAVI and independent variable DBP

Table S 6- test of autocorrelation for the Linear regression, dependent variable Right CAVI and independent variable DBP

Durbin-Watson Statistics

**Order**	**DW**	**Pr < DW**	**Pr > DW**
**1**	1.8595	0.1830	0.8170

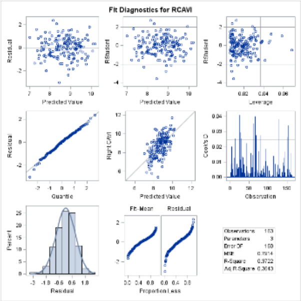

Figure S 4- Fit statistics and assumption checking for the Linear regression, dependent variable Right CAVI and independent variable DBP and age

Table S 7- test of autocorrelation for the Linear regression, dependent variable Right CAVI and independent variable DBP and age

Durbin-Watson Statistics

**Order**	**DW**	**Pr < DW**	**Pr > DW**
**1**	2.0753	0.6799	0.3201

Table S 8- Linear regression analysis for the test of collinearity between the independent variables in the regression models, Right CAVI as the dependent variable and DBP and Age as independent ones

Variable	Parameter Estimate	Standard Error	t Value	Pr > |t|	Tolerance	Variance Inflation
**Intercept**	2.61829	0.71897	3.64	0.0004	.	0
**DBP**	0.02187	0.00672	3.25	0.0014	0.99999	1.00001
**AGE**	0.06814	0.00743	9.17	<.0001	0.99999	1.00001

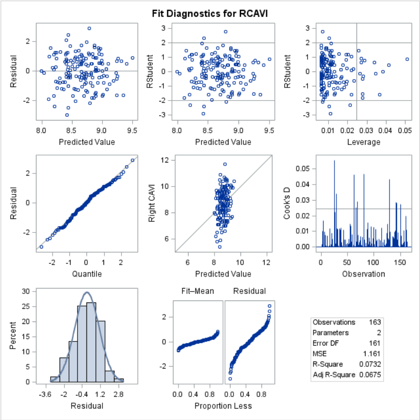

Figure S 5- Fit statistics and assumption checking for the Linear regression, dependent variable Right CAVI and independent variable MBP

Table S 9- test of autocorrelation for the Linear regression, dependent variable Right CAVI and independent variable MBP

Durbin-Watson Statistics

Order	DW	Pr < DW	Pr > DW
**1**	1.8461	0.1614	0.8386

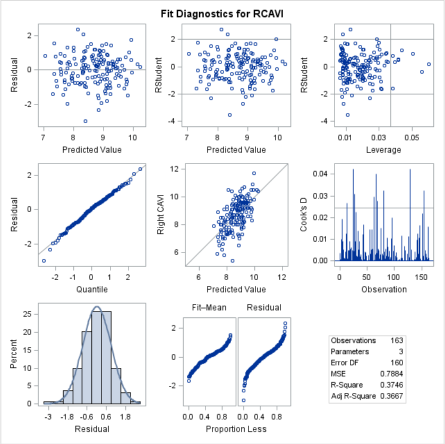

Figure S 6- Fit statistics and assumption checking for the Linear regression, dependent variable Right CAVI and independent variable MBP and age

Table S 10- test of autocorrelation for the Linear regression, dependent variable Right CAVI and independent variable MBP and age

Durbin-Watson Statistics

Order	DW	Pr < DW	Pr > DW
**1**	2.0473	0.6139	0.3861

Table S 11- Linear regression analysis for the test of collinearity between the independent variables in the regression models, Right CAVI as the dependent variable and MBP and Age as independent ones, MBP=Mean Blood Pressure

Variable	Parameter Estimate	Standard Error	t Value	Pr > |t|	Tolerance	Variance Inflation
**Intercept**	2.64996	0.69958	3.79	0.0002	.	0
**MBP**	0.01954	0.00583	3.35	0.0010	0.98830	1.01183
**AGE**	0.06552	0.00746	8.78	<.0001	0.98830	1.01183

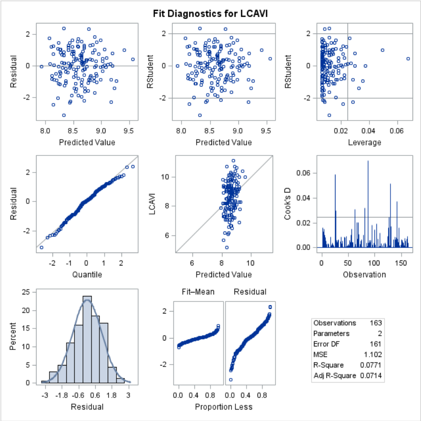

Figure S 7- Fit statistics and assumption checking for the Linear regression, dependent variable Left CAVI and independent variable SBP

Table S 12- test of autocorrelation for the Linear regression, dependent variable Left CAVI and independent variable SBP

Durbin-Watson Statistics

**Order**	**DW**	**Pr < DW**	**Pr > DW**
**1**	1.9306	0.3280	0.6720

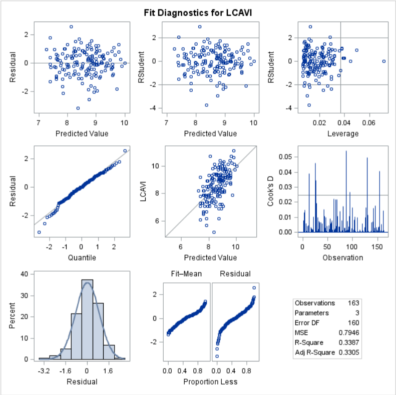

Figure S 8- Fit statistics and assumption checking for the Linear regression, dependent variable Left CAVI and independent variable SBP and age

Table S 13- test of autocorrelation for the Linear regression, dependent variable Left CAVI and independent variable SBP and age

Durbin-Watson Statistics

**Order**	**DW**	**Pr < DW**	**Pr > DW**
**1**	2.0944	0.7235	0.2765

Table S 14- Linear regression analysis for the test of collinearity between the independent variables in the regression models, Left CAVI as the dependent variable and SBP and Age as independent ones

Variable	Parameter Estimate	Standard Error	t Value	Pr > |t|	Tolerance	Variance Inflation
**Intercept**	3.45041	0.64153	5.38	<.0001	.	0
**SBP**	0.01016	0.00413	2.46	0.0150	0.95140	1.05109
**AGE**	0.06074	0.00764	7.96	<.0001	0.95140	1.05109

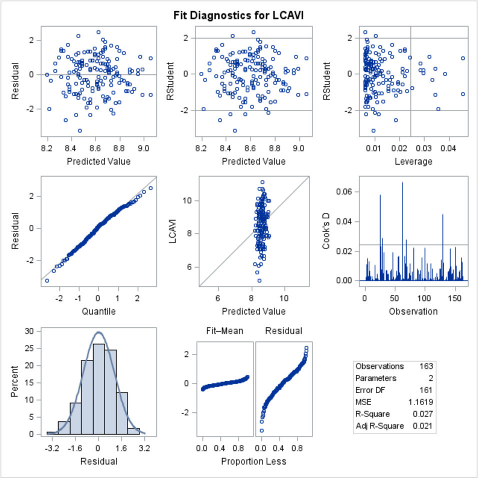

Figure S 9- Fit statistics and assumption checking for the Linear regression, dependent variable Left CAVI and independent variable DBP

Table S 15- test of autocorrelation for the Linear regression, dependent variable Left CAVI and independent variable DBP

Durbin-Watson Statistics

**Order**	**DW**	**Pr < DW**	**Pr > DW**
**1**	1.9706	0.4234	0.5766

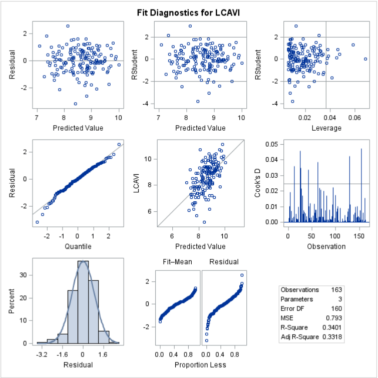

Figure S 10- Fit statistics and assumption checking for the Linear regression, dependent variable Left CAVI and independent variable DBP and age

Table S 16- test of autocorrelation for the Linear regression, dependent variable Left CAVI and independent variable DBP and age

Durbin-Watson Statistics

**Order**	**DW**	**Pr < DW**	**Pr > DW**
**1**	2.1483	0.8249	0.1751

Table S 17- Linear regression analysis for the test of collinearity between the independent variables in the regression models, Left CAVI as the dependent variable and DBP and Age as independent ones

Variable	Parameter Estimate	Standard Error	t Value	Pr > |t|	Tolerance	Variance Inflation
**Intercept**	3.14337	0.71969	4.37	<.0001	.	0
**DBP**	0.01700	0.00672	2.53	0.0124	0.99999	1.00001
**AGE**	0.06482	0.00744	8.71	<.0001	0.99999	1.00001

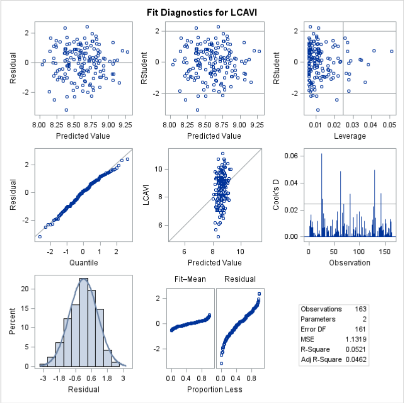

Figure S 11- Fit statistics and assumption checking for the Linear regression, dependent variable Left CAVI and independent variable MBP

Table S 18- test of autocorrelation for the Linear regression, dependent variable Left CAVI and independent variable MBP

Durbin-Watson Statistics

Order	DW	Pr < DW	Pr > DW
**1**	1.9609	0.3998	0.6002

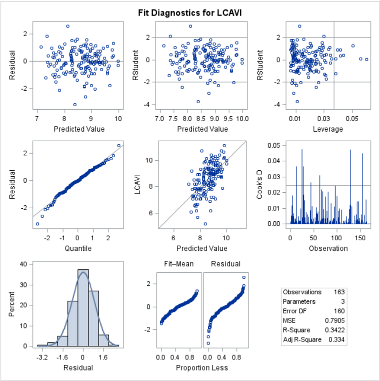

Figure S 12- Fit statistics and assumption checking for the Linear regression, dependent variable Left CAVI and independent variable MBP and age

Table S 19- test of autocorrelation for the Linear regression, dependent variable Left CAVI and independent variable MBP and age

Durbin-Watson Statistics

Order	DW	Pr < DW	Pr > DW
**1**	2.1261	0.7864	0.2136

Table S 20- Linear regression analysis for the test of collinearity between the independent variables in the regression models, Left CAVI as the dependent variable and MBP and Age as independent ones

Variable	Parameter Estimate	Standard Error	t Value	Pr > |t|	Tolerance	Variance Inflation
**Intercept**	3.15371	0.70049	4.50	<.0001	.	0
**MBP**	0.01535	0.00583	2.63	0.0093	0.98830	1.01183
**AGE**	0.06276	0.00747	8.40	<.0001	0.98830	1.01183

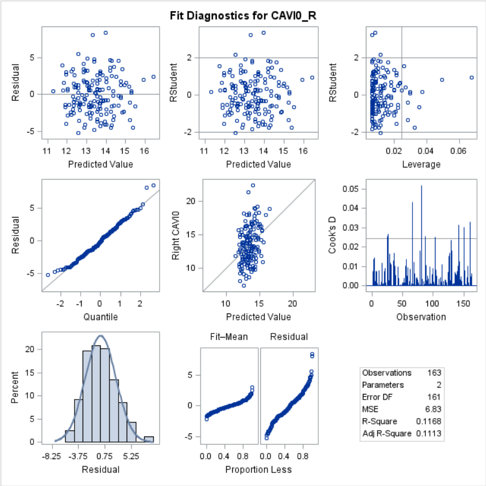

Figure S 13- Fit statistics and assumption checking for the Linear regression, dependent variable Right $CAVI_0$ and independent variable SBP

Table S 21- test of autocorrelation for the Linear regression, dependent variable Right $CAVI_0$ and independent variable SBP

Durbin-Watson Statistics

Order	DW	Pr < DW	Pr > DW
**1**	1.8613	0.1869	0.8131

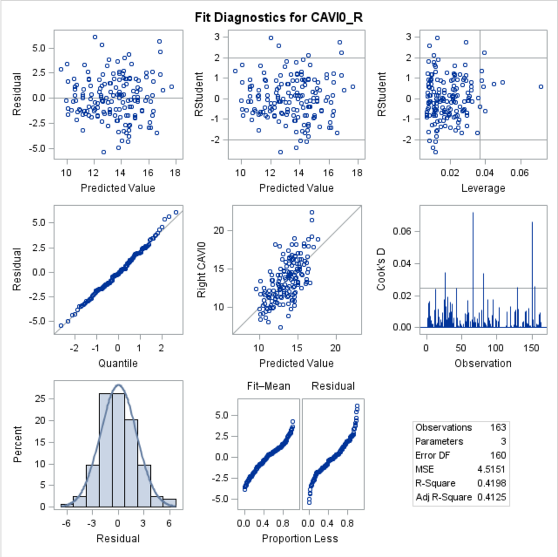

Figure S 14- Fit statistics and assumption checking for the Linear regression, dependent variable Right $CAVI_0$ and independent variable SBP and age

Table S 22- test of autocorrelation for the Linear regression, dependent variable Right $CAVI_0$ and independent variable SBP and age

Durbin-Watson Statistics

Order	DW	Pr < DW	Pr > DW
**1**	2.1264	0.7875	0.2125

Table S 23- Linear regression analysis for the test of collinearity between the independent variables in the regression models, Right CAVI0 as the dependent variable and SBP and Age as independent ones, SBP=Systolic Blood Pressure

Variable	Parameter Estimate	Standard Error	t Value	Pr > |t|	Tolerance	Variance Inflation
**Intercept**	-1.57302	1.52925	-1.03	0.3052	.	0
**SBP**	0.03468	0.00985	3.52	0.0006	0.95140	1.05109
**AGE**	0.16636	0.01820	9.14	<.0001	0.95140	1.05109

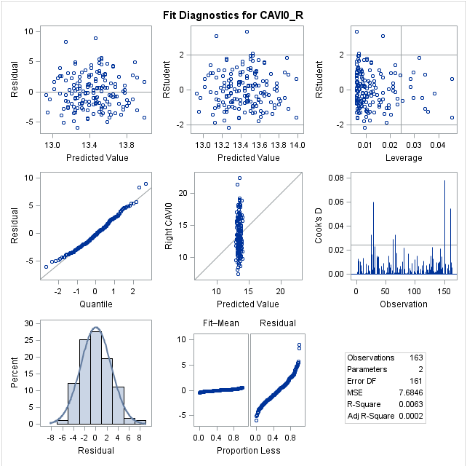

Figure S 15- Fit statistics and assumption checking for the Linear regression, dependent variable Right $CAVI_0$ and independent variable DBP

Table S 24- test of autocorrelation for the Linear regression, dependent variable Right $CAVI_0$ and independent variable DBP

Durbin-Watson Statistics

Order	DW	Pr < DW	Pr > DW
**1**	1.8990	0.2575	0.7425

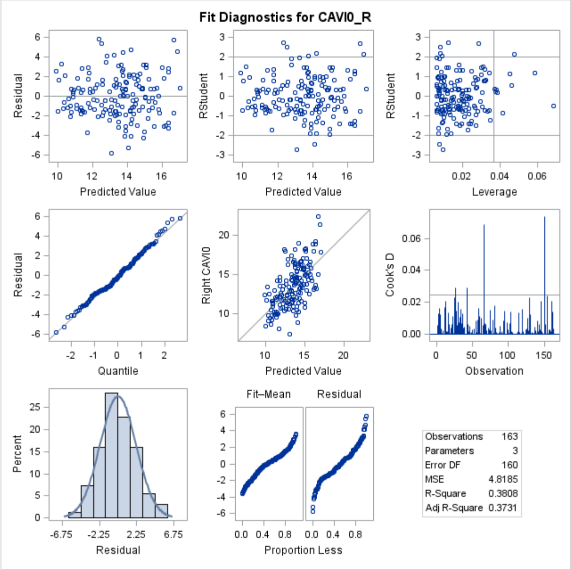

Figure S 16- Fit statistics and assumption checking for the Linear regression, dependent variable Right CAVI$_0$ and independent variable DBP and age

Table S 25- test of autocorrelation for the Linear regression, dependent variable Right CAVI$_0$ and independent variable DBP and age

Durbin-Watson Statistics			
**Order**	**DW**	**Pr < DW**	**Pr > DW**
**1**	2.1625	0.8475	0.1525

Table S 26- Linear regression analysis for the test of collinearity between the independent variables in the regression models, Right CAVI0 as the dependent variable and DBP and Age as independent ones, DBP=Diastolic Blood Pressure

Variable	Parameter Estimate	Standard Error	t Value	Pr > |t|	Tolerance	Variance Inflation
**Intercept**	0.38898	1.77407	0.22	0.8267	.	0
**DBP**	0.02061	0.01658	1.24	0.2155	0.99999	1.00001
**AGE**	0.18041	0.01834	9.84	<.0001	0.99999	1.00001

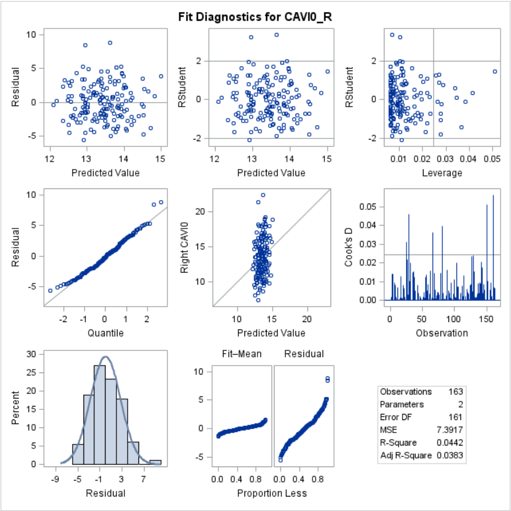

Figure S 17- Fit statistics and assumption checking for the Linear regression, dependent variable Right $CAVI_0$ and independent variable MBP

Table S 27- test of autocorrelation for the Linear regression, dependent variable Right $CAVI_0$ and independent variable MBP

Durbin-Watson Statistics

Order	DW	Pr < DW	Pr > DW
1	1.8954	0.2506	0.7494

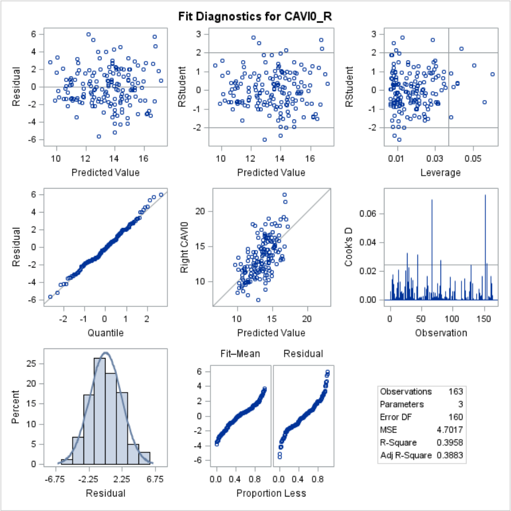

Figure S 18- Fit statistics and assumption checking for the Linear regression, dependent variable Right $CAVI_0$ and independent variable MBP and age

Table S 28- test of autocorrelation for the Linear regression, dependent variable Right $CAVI_0$ and independent variable MBP and age

Durbin-Watson Statistics			
**Order**	**DW**	**Pr < DW**	**Pr > DW**
**1**	2.1564	0.8385	0.1615

Table S 29- Linear regression analysis for the test of collinearity between the independent variables in the regression models, Right CAVI0 as the dependent variable and MBP and Age as independent ones, MBP=Mean Blood Pressure

Variable	Parameter Estimate	Standard Error	t Value	Pr > |t|	Tolerance	Variance Inflation
**Intercept**	-0.92004	1.70839	-0.54	0.5910	.	0
**MBP**	0.03355	0.01423	2.36	0.0196	0.98830	1.01183
**AGE**	0.17584	0.01822	9.65	<.0001	0.98830	1.01183

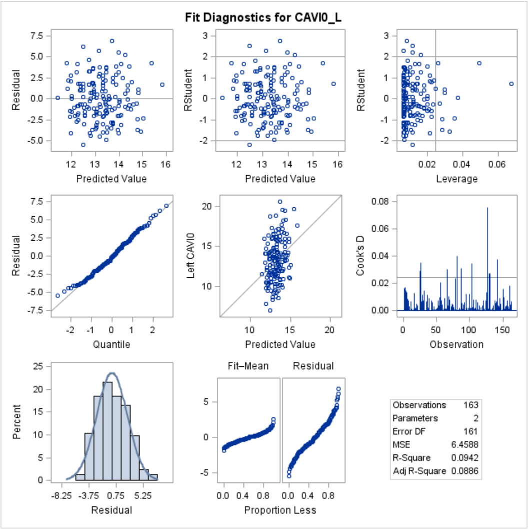

Figure S 19- Fit statistics and assumption checking for the Linear regression, dependent variable Left $CAVI_0$ and independent variable SBP

Table S 30- test of autocorrelation for the Linear regression, dependent variable Left $CAVI_0$ and independent variable SBP

Durbin-Watson Statistics

Order	DW	Pr < DW	Pr > DW
**1**	1.9868	0.4657	0.5343

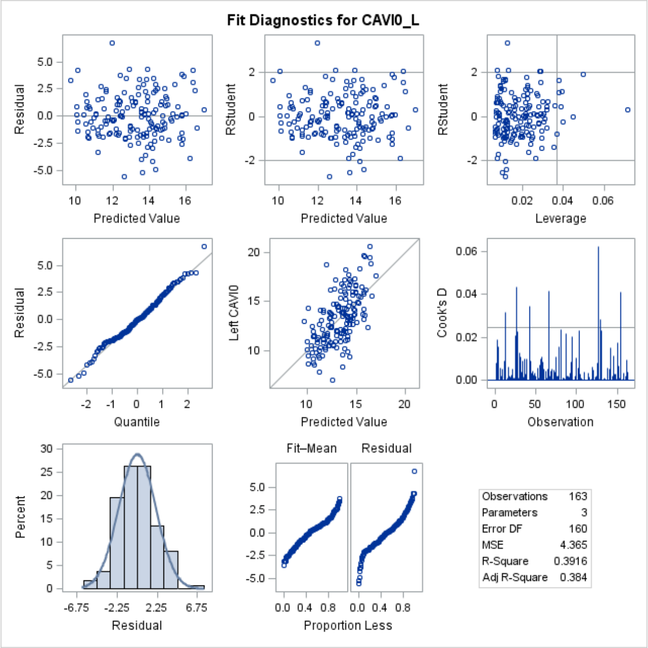

Figure S 20- Fit statistics and assumption checking for the Linear regression, dependent variable Right $CAVI_0$ and independent variable SBP and age

Table S 31- test of autocorrelation for the Linear regression, dependent variable Left CAVI0 and independent variable SBP and age

Durbin-Watson Statistics

Order	DW	Pr < DW	Pr > DW
**1**	2.1975	0.8952	0.1048

Table S 32- Linear regression analysis for the test of collinearity between the independent variables in the regression models, Left CAVI0 as the dependent variable and SBP and Age as independent ones, SBP=Systolic Blood Pressure

Variable	Parameter Estimate	Standard Error	t Value	Pr > |t|	Tolerance	Variance Inflation
**Intercept**	-0.41034	1.50361	-0.27	0.7853	.	0
**sbp**	0.02813	0.00968	2.90	0.0042	0.95140	1.05109
**AGE**	0.15828	0.01790	8.84	<.0001	0.95140	1.05109

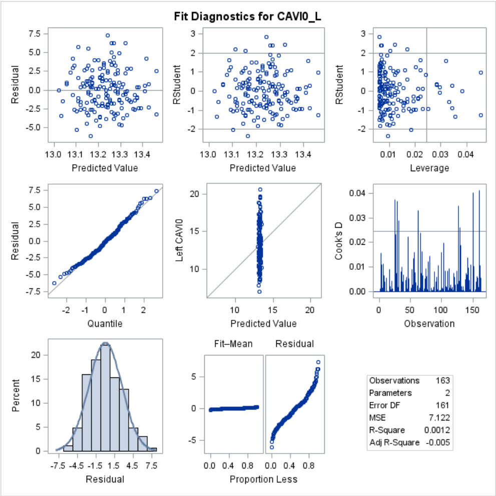

Figure S 21- Fit statistics and assumption checking for the Linear regression, dependent variable Right CAVI0 and independent variable DBP

Table S 33- test of autocorrelation for the Linear regression, dependent variable Left $CAVI_0$ and independent variable DBP

Durbin-Watson Statistics

Order	DW	Pr < DW	Pr > DW
1	2.0163	0.5394	0.4606

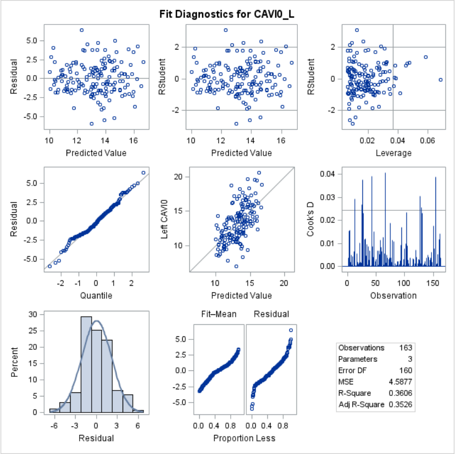

Figure S 22- Fit statistics and assumption checking for the Linear regression, dependent variable Right $CAVI_0$ and independent variable DBP and age

Table S 34- test of autocorrelation for the Linear regression, dependent variable Left $CAVI_0$ and independent variable DBP and age

Durbin-Watson Statistics

Order	DW	Pr < DW	Pr > DW
1	2.2235	0.9218	0.0782

Table S 35- Linear regression analysis for the test of collinearity between the independent variables in the regression models, Left CAVI0 as the dependent variable and DBP and Age as independent ones, DBP=Diastolic Blood Pressure

Variable	Parameter Estimate	Standard Error	t Value	Pr > |t|	Tolerance	Variance Inflation
**Intercept**	1.86505	1.73107	1.08	0.2829	.	0
**dbp**	0.00822	0.01617	0.51	0.6122	0.99999	1.00001
**AGE**	0.16971	0.01790	9.48	<.0001	0.99999	1.00001

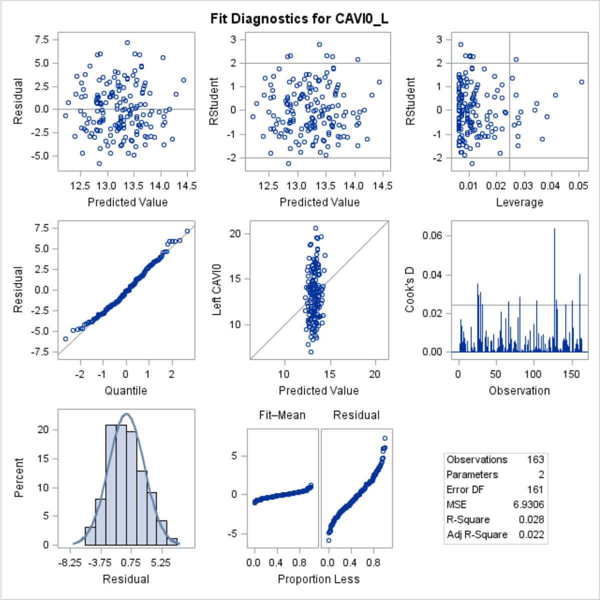

Figure S 23- Fit statistics and assumption checking for the Linear regression, dependent variable Right $CAVI_0$ and independent variable MBP

Table S 36- test of autocorrelation for the Linear regression, dependent variable Left CAVI0 and independent variable MBP

Durbin-Watson Statistics

Order	DW	Pr < DW	Pr > DW
**1**	2.0162	0.5397	0.4603

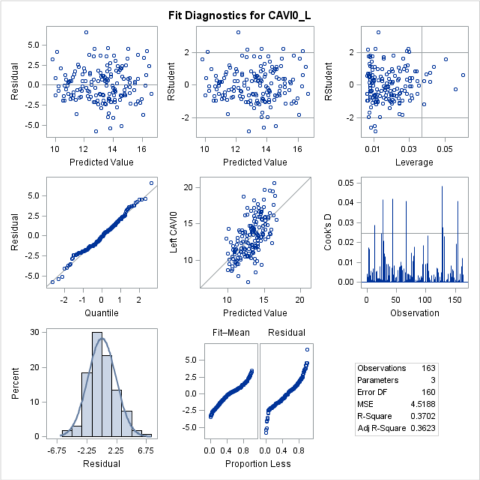

Figure S 24- Fit statistics and assumption checking for the Linear regression, dependent variable Right $CAVI_0$ and independent variable MBP and age

Table S 37- test of autocorrelation for the Linear regression, dependent variable Left CAVI0 and independent variable MBP and age

Durbin-Watson Statistics

Order	DW	Pr < DW	Pr > DW
**1**	2.2213	0.9201	0.0799

Table S 38- Linear regression analysis for the test of collinearity between the independent variables in the regression models, Left CAVI0 as the dependent variable and MBP and Age as independent ones, MBP=Mean Blood Pressure

Variable	Parameter Estimate	Standard Error	t Value	Pr > |t|	Tolerance	Variance Inflation
**Intercept**	0.49781	1.67485	0.30	0.7667	.	0
**mbp**	0.02293	0.01395	1.64	0.1022	0.98830	1.01183
**AGE**	0.16657	0.01786	9.32	<.0001	0.98830	1.01183

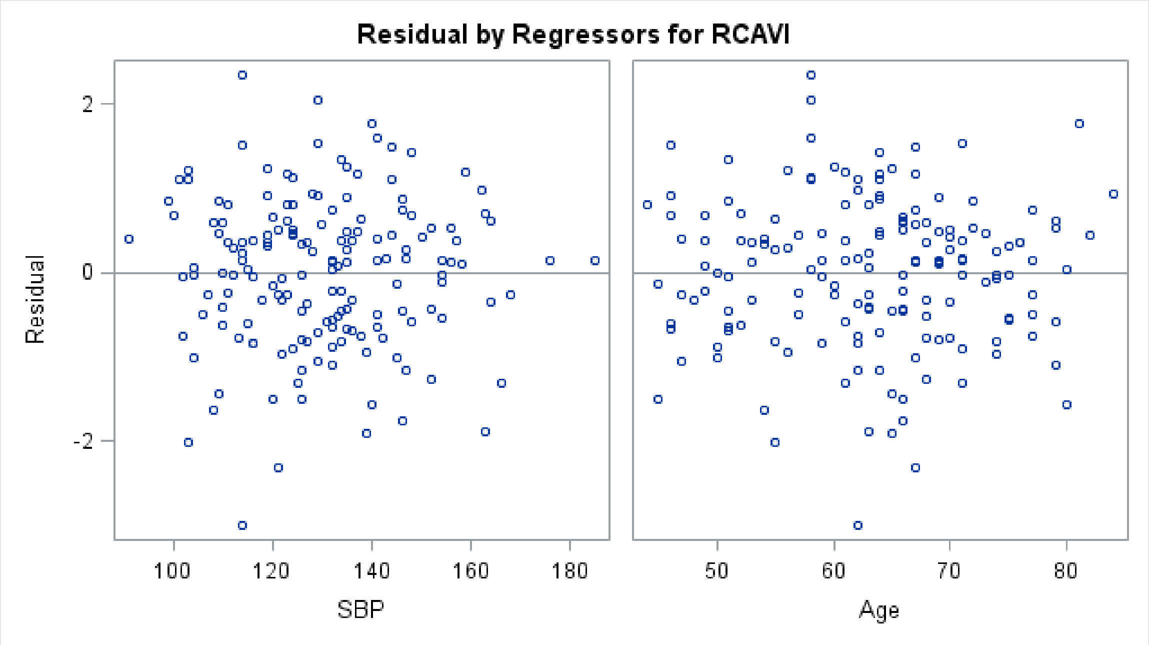

Figure S 25- Test for the independence of errors assumption, RCAVI=right CAVI, SBP=Systolic Blood Pressure

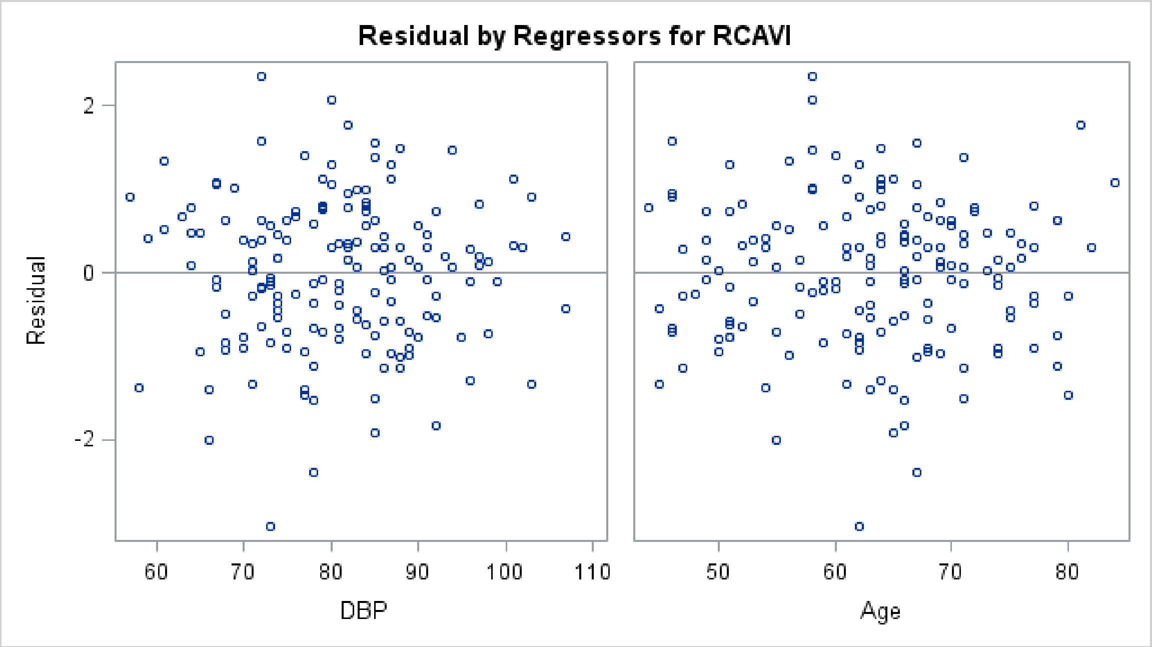

Figure S 26- Test for the independence of errors assumption, RCAVI=right CAVI, DBP=Diastolic Blood Pressure

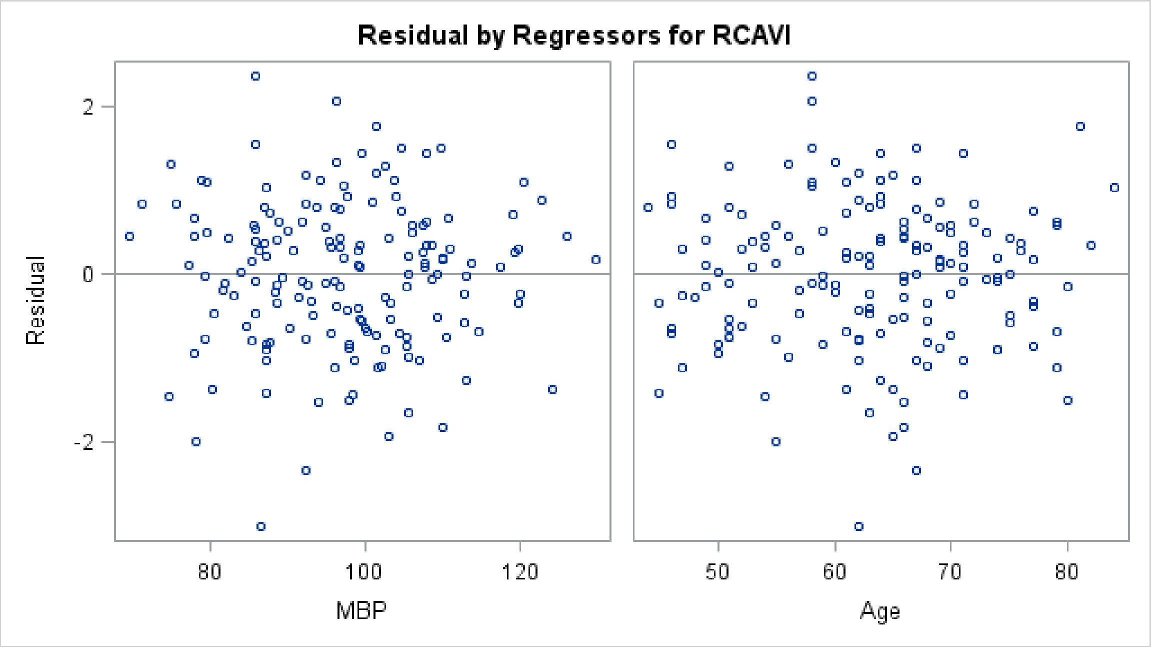

Figure S 27- Test for the independence of errors assumption, RCAVI=right CAVI, MBP=Mean Blood Pressure

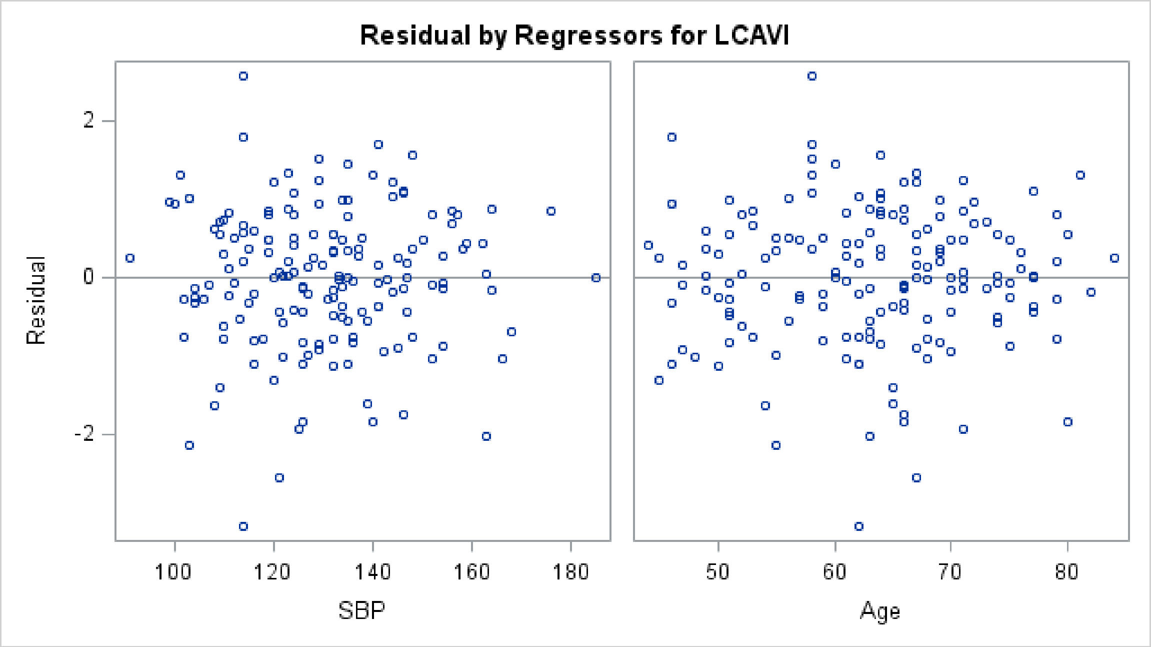

Figure S 28- Test for the independence of errors assumption, LCAVI=Left CAVI, SBP=Systolic Blood Pressure

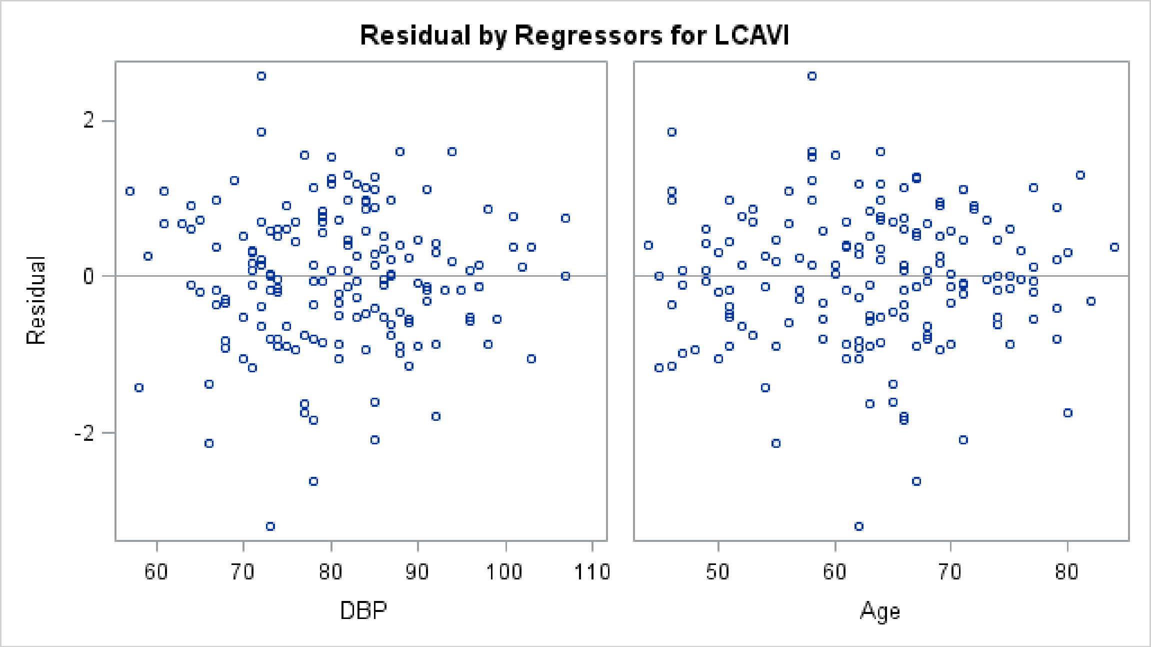

Figure S 29- Test for the independence of errors assumption, LCAVI=Left CAVI, DBP=Diastolic Blood Pressure

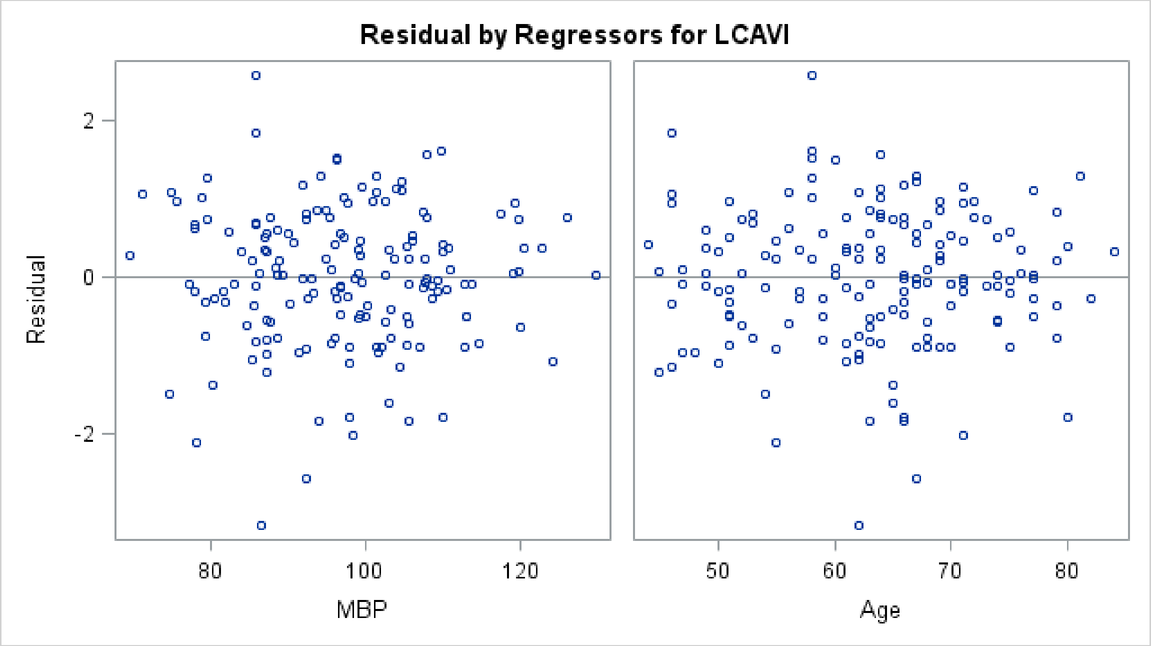

Figure S 30- Test for the independence of errors assumption, LCAVI=Left CAVI, MBP=Mean Blood Pressure

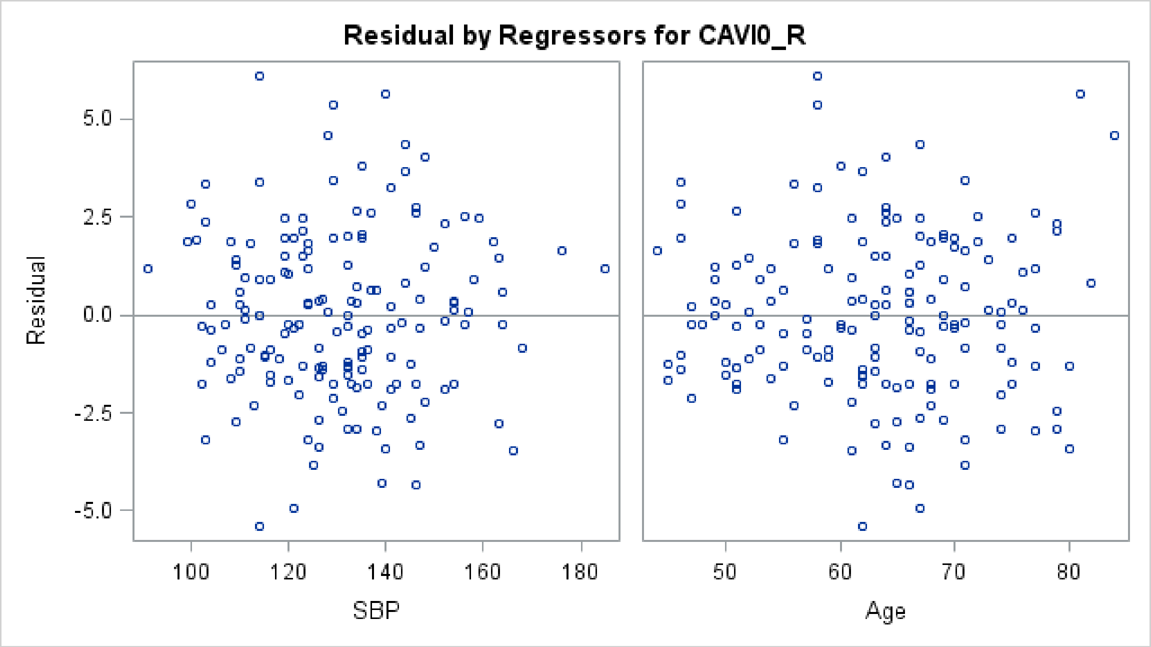

Figure S 31- Test for the independence of errors assumption, CAVI0_R=Right CAVI, SBP=Systolic Blood Pressure

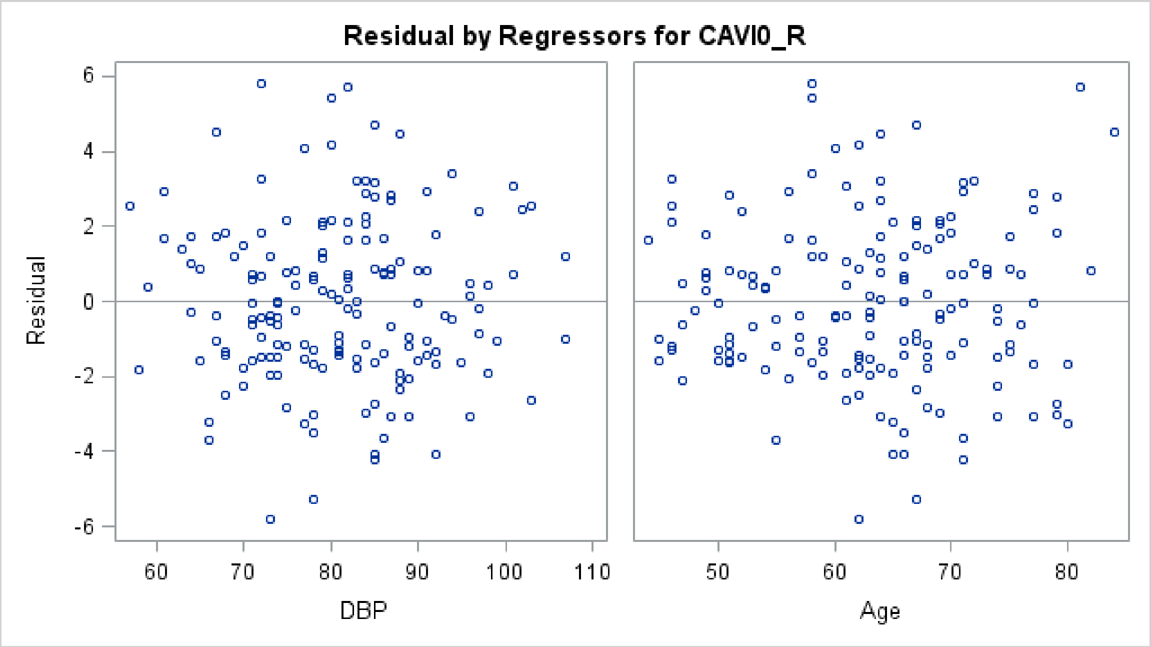

Figure S 32- Test for the independence of errors assumption, CAVI0_R=Right CAVI, DBP=Diastolic Blood Pressure

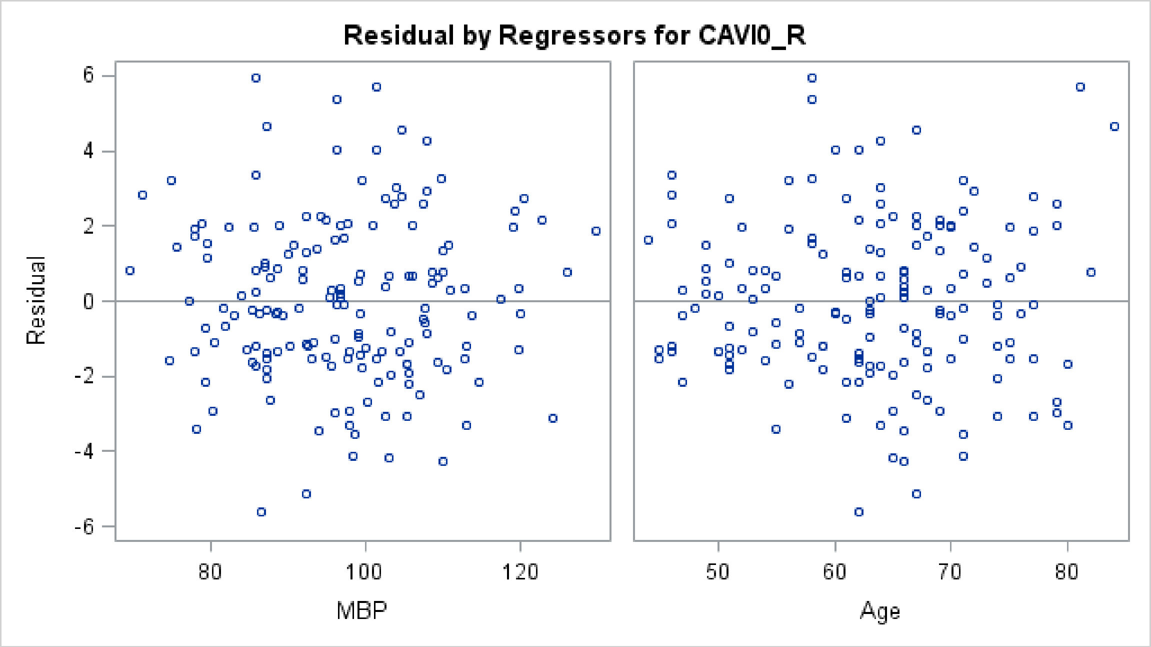

Figure S 33- Test for the independence of errors assumption, CAVI0_R=Right CAVI, MBP=Mean Blood Pressure
